# Interaction of periodontitis and orthodontic tooth movement—an in vitro and in vivo study

**DOI:** 10.1007/s00784-021-03988-4

**Published:** 2021-05-23

**Authors:** Birgit Rath-Deschner, Andressa V. B. Nogueira, Svenja Beisel-Memmert, Marjan Nokhbehsaim, Sigrun Eick, Joni A. Cirelli, James Deschner, Andreas Jäger, Anna Damanaki

**Affiliations:** 1 Department of Orthodontics, Center of Dento-Maxillo-Facial Medicine, University of Bonn, Welschnonnenstrasse 17, 53111 Bonn, Germany; 2 Department of Periodontology and Operative Dentistry, University Medical Center of the Johannes Gutenberg University, Mainz, Germany; 3 Section of Experimental Dento-Maxillo-Facial Medicine, Center of Dento-Maxillo-Facial Medicine, University of Bonn, Bonn, Germany; 4 Department of Periodontology, Laboratory for Oral Microbiology, University of Bern, Bern, Switzerland; 5 Department of Diagnosis and Surgery, School of Dentistry at Araraquara, Sao Paulo State University, UNESP, Araraquara, Brazil

**Keywords:** Orthodontic tooth movement, Periodontitis, *Fusobacterium nucleatum*, Periodontium

## Abstract

**Objectives:**

The aim of this in vitro and in vivo study was to investigate the interaction of periodontitis and orthodontic tooth movement on interleukin (IL)-6 and C-X-C motif chemokine 2 (CXCL2).

**Materials and methods:**

The effect of periodontitis and/or orthodontic tooth movement (OTM) on alveolar bone and gingival IL-6 and CXCL2 expressions was studied in rats by histology and RT-PCR, respectively. The animals were assigned to four groups (control, periodontitis, OTM, and combination of periodontitis and OTM). The IL-6 and CXCL2 levels were also studied in human gingival biopsies from periodontally healthy and periodontitis subjects by RT-PCR and immunohistochemistry. Additionally, the synthesis of IL-6 and CXCL2 in response to the periodontopathogen *Fusobacterium nucleatum* and/or mechanical strain was studied in periodontal fibroblasts by RT-PCR and ELISA.

**Results:**

Periodontitis caused an increase in gingival levels of IL-6 and CXCL2 in the animal model. Moreover, orthodontic tooth movement further enhanced the bacteria-induced periodontal destruction and gingival IL-6 gene expression. Elevated IL-6 and CXCL2 gingival levels were also found in human periodontitis. Furthermore, mechanical strain increased the stimulatory effect of *F. nucleatum* on IL-6 protein in vitro.

**Conclusions:**

Our study suggests that orthodontic tooth movement can enhance bacteria-induced periodontal inflammation and thus destruction and that IL-6 may play a pivotal role in this process.

**Clinical relevance:**

Orthodontic tooth movement should only be performed after periodontal therapy. In case of periodontitis relapse, orthodontic therapy should be suspended until the periodontal inflammation has been successfully treated and thus the periodontal disease is controlled again.

## Introduction

The aim of orthodontic therapy is to identify and treat tooth and jaw misalignments early in life. Although orthodontic therapy is mostly started in children and adolescents, the proportion of adults seeking orthodontic treatment is steadily increasing in our aging society [1]. However, advancing age is also associated with other oral diseases, such as periodontitis [2]. Periodontitis is characterized by irreversible loss of the periodontium and, if left untreated, can lead to tooth loss, reduced masticatory function, and psychological and general medical problems [3]. Orthodontic tooth movement also results in the breakdown of structures of the periodontium. However, this controlled degradation is limited in time and location and is accompanied by the simultaneous formation of new periodontal structures [4]. Overall, this process is therefore a remodeling of the periodontal structures, which is usually not associated with a net loss of periodontal bone and attachment.

Patients with severely advanced periodontitis (stage IV), who have been successfully treated, often suffer from the consequences of periodontitis such as tooth migration or flaring of the maxillary anterior teeth [5, 6]. A combined orthodontic-periodontal therapy may therefore be necessary for a functionally and aesthetically satisfactory treatment outcome. Some clinical studies have been dedicated to such a combination therapy and have proven that treated periodontitis patients can be successfully managed orthodontically afterwards [7–10]. However, there is a consensus among orthodontists and periodontists that orthodontic therapy is not indicated if periodontal disease has not been successfully treated or if periodontitis relapses, because otherwise aggravation of bacteria-induced periodontal inflammation and destruction may occur [11].

The mechanisms by which periodontal pathogenic bacteria, such as *Fusobacterium nucleatum*, interact with mechanical/orthodontic forces are incompletely understood. *F. nucleatum* is a gram-negative and anaerobic microorganism, which has an important function as a so-called bridging bacterium between early and late colonizers in biofilm formation. *F. nucleatum* plays a significant etiopathogenetic role in gingivitis and periodontitis [12–16]. Previous studies by our research group have shown that mechanical/orthodontic forces can regulate the bacteria-induced levels of some pro-inflammatory and anti-apoptotic molecules [17–19]. The synthesis of some of these molecules during periodontal inflammation is further enhanced by mechanical/orthodontic forces, whereas other molecules tend to be downregulated [17–19]. Overall, these studies show that mechanical/orthodontic forces can influence the host immunoinflammatory response to periodontal bacteria.

Two molecules that are important in such immunoinflammatory processes are interleukin (IL)-6 and the C-X-C motif chemokine 2 (CXCL2). IL-6 plays a key role in immunoinflammatory processes, links innate and acquired immunity, and is considered a stimulator of acute phase protein synthesis and a lymphocyte stimulating factor [20, 21]. CXCL2, also known as macrophage inflammatory protein 2, is produced by monocytes, macrophages, and other cells at sites of inflammation; is elevated in many inflammatory and autoimmune diseases; and exerts a chemotactic effect on neutrophil granulocytes [22, 23].

The aim of this in vitro and in vivo study was to investigate the single and combined effects of periodontitis and orthodontic tooth movement on IL-6 and CXCL2. Our hypothesis was that orthodontic tooth movement may enhance the synthesis of these two molecules in periodontal inflammation and destruction.

## Materials and methods

### Human gingival biopsies from periodontally healthy and periodontitis sites

Inflamed gingival tissue from periodontitis patients (n = 7) became available during tooth extractions performed for periodontal reasons in the Department of Oral Surgery at the University of Bonn. Healthy gingiva (n = 7), on the other hand, could be obtained for the study during wisdom tooth surgery and tooth extractions for orthodontic reasons. The approval of the ethics committee of the University of Bonn (#043/11) had been obtained in advance. The patients or their parents or legal representatives had also given written informed consent. Gingiva was only taken from those patients who did not suffer from systemic diseases and were non-smokers. Gingival sites with a gingival index = 0 (no clinical inflammation), periodontal probing depth ≤ 3 mm, no clinical attachment loss, and no radiographic bone loss were classified as periodontally healthy, while gingival sites with a gingival index > 1, periodontal pocket depth ≥ 5 mm, clinical attachment loss ≥ 3 mm, and radiographic bone loss were classified as periodontitis. The gingival biopsies were then either stored at −80 °C for later gene expression analysis using an RNA stabilization reagent (RNAlater, Qiagen, Hilden, Germany) or stored directly in formaldehyde for later immunohistochemical analysis.

### Quantitative real-time polymerase chain reaction

For the subsequent real-time polymerase chain reaction (PCR), total RNA was extracted from the gingival biopsies and periodontal cells (see below) using the RNeasy Mini Kit (Qiagen, Hilden, Germany) according to the manufacturer’s protocol. The RNA concentrations were then measured using the NanoDrop ND-2000 spectrophotometer (Thermo Fisher Scientific, Wilmington, DE, USA), and 500 ng of total RNA was reverse transcribed using the iScript™ Select cDNA Synthesis Kit (Bio-Rad Laboratories, Munich, Germany) at 42 °C for 90 min followed by 85 °C for 5 min, according to the manufacturer’s protocol. The IL-6 and CXCL2 gene expressions were then determined using QuantiTect primers (Qiagen), SYBR Green QPCR Master Mix (Bio-Rad), and the iCycler iQ™ Real-Time PCR Detection System (Bio-Rad). The amplification was performed as follows: initial denaturation at 95 °C for 5 min, followed by 40 cycles of denaturation at 95 °C for 10 s and combined annealing/extension at 60 °C for 30 s. Glyceraldehyde-3-phosphate dehydrogenase was used as the housekeeping gene.

### Immunohistochemical analysis

After 2 days of fixation in 4% formaldehyde (Merck, Darmstadt, Germany), the human gingival biopsies were hydrated and dehydrated in an ascending ethanol series (AppliChem, Darmstadt, Germany) and embedded in paraffin (McCormick Scientific, Richmond, IL, USA). Sections of 2.5-μm thickness were mounted on glass slides (Engelbrecht, Edermünde, Germany) and dried overnight at 37 °C. For immunohistochemical analysis, the sections were deparaffinized and rehydrated. Subsequently, endogenous peroxidase was blocked with 0.3% methanol (Merck)/30% H_2_O_2_ (Merck) solution for 10 min. IL-6 and CXCL2 were detected by incubation with a mouse monoclonal anti-IL-6 antibody (ab9324, 1:100; Abcam, Berlin, Germany) and a rabbit polyclonal anti-CXCL2-antibody (TA328332, OriGene, Rockville, MD, USA) at 4 °C overnight. Sections were then washed, followed by incubation with a goat anti-mouse or anti-rabbit IgG-HRP secondary antibody (Dako, Hamburg, Germany) for 30 min at room temperature. Visualization of peroxidase activity was performed with 3,3′-diaminobenzidine chromogen (Thermo Fisher Scientific), followed by rinsing and counterstaining with Mayer’s hematoxylin (Merck) for 1 min. Using an Axioskop 2 microscope and AxioVision 4.7 software (Carl Zeiss, Oberkochen, Germany), photographs were taken and subsequently analyzed.

### Experimental periodontitis and/or orthodontic tooth movement

**Fig. 1:**
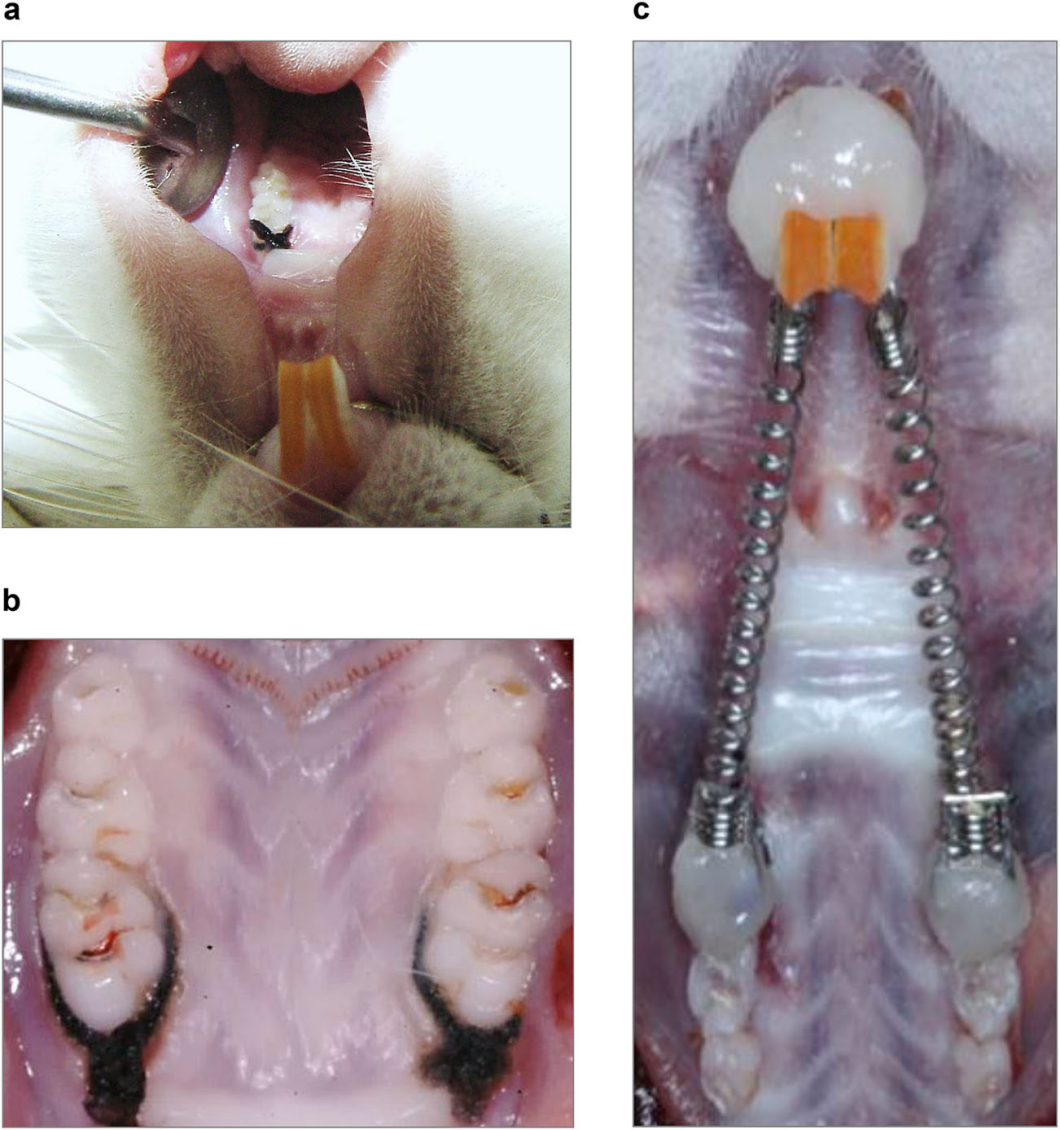
Experimental periodontitis and/or orthodontic tooth movement in rats. An animal model was used to study the influence of periodontitis and/or orthodontic tooth movement on periodontal tissues. Experimental periodontitis was induced by cotton ligatures, which were tied around the cervical region of the maxillary first molars (**a** and **b**). Orthodontic tooth movement was accomplished by a closed nickel-titanium coil spring placed between the maxillary first molars and central incisors (**c**)

An animal model was used to study the influence of periodontitis and orthodontic tooth movement on periodontal tissues. The experiments were performed according to the ARRIVE guidelines (Animal Research: Reporting of In Vivo Experiments) and approved by the Ethics Committee for Animal Experiments at the School of Dentistry in Araraquara, São Paulo State University-UNESP (protocol number: 23/2012). Adult Holtzman rats of 300 g were kept in the animal facility of the university and provided with standard laboratory chow and water ad libitum. Rats were randomly assigned to the following groups: (a) untreated control, (b) periodontitis, (c) orthodontic tooth movement, (d) combination of periodontitis and orthodontic tooth movement. The animals (n = 4 rats/group) were treated under general anesthesia consisting of ketamine chlorohydrate 10% (0.08 ml/100 g body weight) and xylazine chlorohydrate 2% (0.04 ml/100 g body weight) injected intramuscularly. Experimental periodontitis was induced by ligatures. Briefly, the cotton ligatures were tied around the cervical region of the maxillary first molars, with the node placed mesially (Fig. 1a, b). Orthodontic tooth movement was achieved by a closed nickel-titanium coil spring (Sentalloy^®^, GAC, Dentsply) placed between the maxillary first molars and central incisors, delivering a relatively constant force of 25 g. The force was applied between the maxillary first molars and central incisors. On the maxillary first molars, the spring was attached with composite resin (Fig. 1c). Grooves had been prepared on the maxillary central incisors to prevent displacement of the 0.20-mm-thick steel wire (CrNi, 55.01.208, Morelli, Brazil). Finally, the wire in the groove was covered with a thin layer of composite resin (Fig. 1c). Occlusal interference was avoided by extracting the mandibular first molars. After 8 days, the animals were sacrificed. The gingiva around the upper first molars was gently removed for subsequent gene expression analyses by real-time PCR (see above). The maxillae were fixed in 4% paraformaldehyde for 2 days. Afterwards, the samples were decalcified in EDTA (10%, 0.5 M) for 3 months and then embedded in paraffin and stored for further histological examination.

### Hematoxylin and eosin staining and stereometric analysis

Morphometric changes were assessed in four hemimaxillae obtained from four animals in each group. Serial parasagittal sections (4 μm) were mounted on slides and stained with hematoxylin and eosin (H&E). Three sections were selected from each tooth. Histometric analysis consisted of determining the area of unmineralized tissue in the furcation region. In the furcation region, the delimitation of the area was according to Duarte et al. [24]. The area of interest included a 1,000-μm zone under the furcation roof in the interradicular region of the maxillary first molar. The total area of the interradicular region and the area of bony mineralized tissue were measured. To determine the area of unmineralized tissue, the bone tissue area was subtracted from the total area. The area of unmineralized tissue was then expressed as a percentage of the total area. The analysis was performed by a blinded and calibrated examiner using a Leica DMLS microscope (Leica microsystems, Wetzlar, Germany; ×200 magnification). Areas of interest were selected and photographed using a Leica DFC 300 FX digital camera (Leica microsystems). The photographs taken were archived in TIFF format. Histometric analysis was performed using the Image J 1.37b image analysis system (National Institutes of Health, Bethesda, MD, USA).

### Cell culture and stimulation

Human periodontal ligament fibroblasts were obtained from periodontally healthy teeth that became available during wisdom tooth surgery or tooth extractions for orthodontic reasons. Approval from the ethics committee of the University of Bonn was available (#043/11), and patients or their parents or legal representatives had given written informed consent. Periodontal fibroblasts were obtained from the middle third of the root surface and cultured in Dulbecco’s minimal essential medium (DMEM, Invitrogen, Karlsruhe, Germany) supplemented with 10% fetal bovine serum (FBS, Invitrogen), 100 U/mL penicillin, and 100 μg/mL streptomycin (Invitrogen) at 37 °C in a humidified atmosphere of 5% CO_2_. Periodontal fibroblasts were used from 3^rd^ to 5^th^ passage. Before the experiments, the FBS concentration was reduced to 1% and the medium was changed every 2 days during the experiments. Cells were incubated with *F. nucleatum* ATCC 25586 (OD_660_: 0.05) to simulate periodontal inflammation in vitro. Bacteria were suspended in phosphate-buffered saline (OD_660_=1.0, equivalent to 1.2×10^9^ bacterial cells/mL) and subjected to ultrasonic sonication twice (160 W for 15 min). To simulate orthodontic tooth movement in vitro, periodontal fibroblasts were subjected to constant tensile strain (CTS) for up to 2 days using an established cell stretching device, as in previous studies [17–19]. In addition, cells were also exposed to *F. nucleatum* and CTS simultaneously. Untreated cells served as controls. Cells were also pre-incubated with a specific inhibitor against mitogen-activated protein kinase kinase (MEK) 1/2 (U0126, 10 μM; Calbiochem, San Diego, CA, USA), 1 h prior to the start of the experiments. At the end of the experiments, the RNA of the cells was obtained for the subsequent analysis of gene expressions by real-time PCR (see above) and the cell supernatants were collected for the further measurement of the protein levels.

### Enzyme-linked immunosorbent assay

Cell supernatants were obtained for the determination of IL-6 and CXCL2 protein levels. Analysis was performed using a microplate reader (PowerWave X, BioTek Instruments, Winooski, VT, USA), which measured absorbance at 450 nm, and commercially available enzyme-linked immunosorbent assay (ELISA) kits for IL-6 (R&D Systems, Abingdon, UK) and CXCL2 (Abnova, Taipei, Taiwan) according to the manufacturer’s instructions. An automated cell counter (Moelab, Hilden, Germany) was used to determine cell counts, which were used to normalize the measured protein concentrations.

### Statistical analysis

Statistical analysis was performed using IBM SPSS Statistics software (version 22, IBM SPSS, Chicago, IL, USA). To test for significant differences between the groups of cell experiments and human and rat gingival biopsies, the t-test or Mann-Whitney U test was used according to the result of the Kolmogorov-Smirnov test for normal distribution. For multiple comparisons, the Bonferroni correction was applied. For the analysis of the area of unmineralized tissue in the animal experiment, the Shapiro-Wilk test and ANOVA followed by Tukey’s post hoc test were performed. Differences between groups were considered significant at p<0.05.

## Results

### Gene expression and protein levels of IL-6 and CXCL2 in human gingiva

**Fig. 2:**
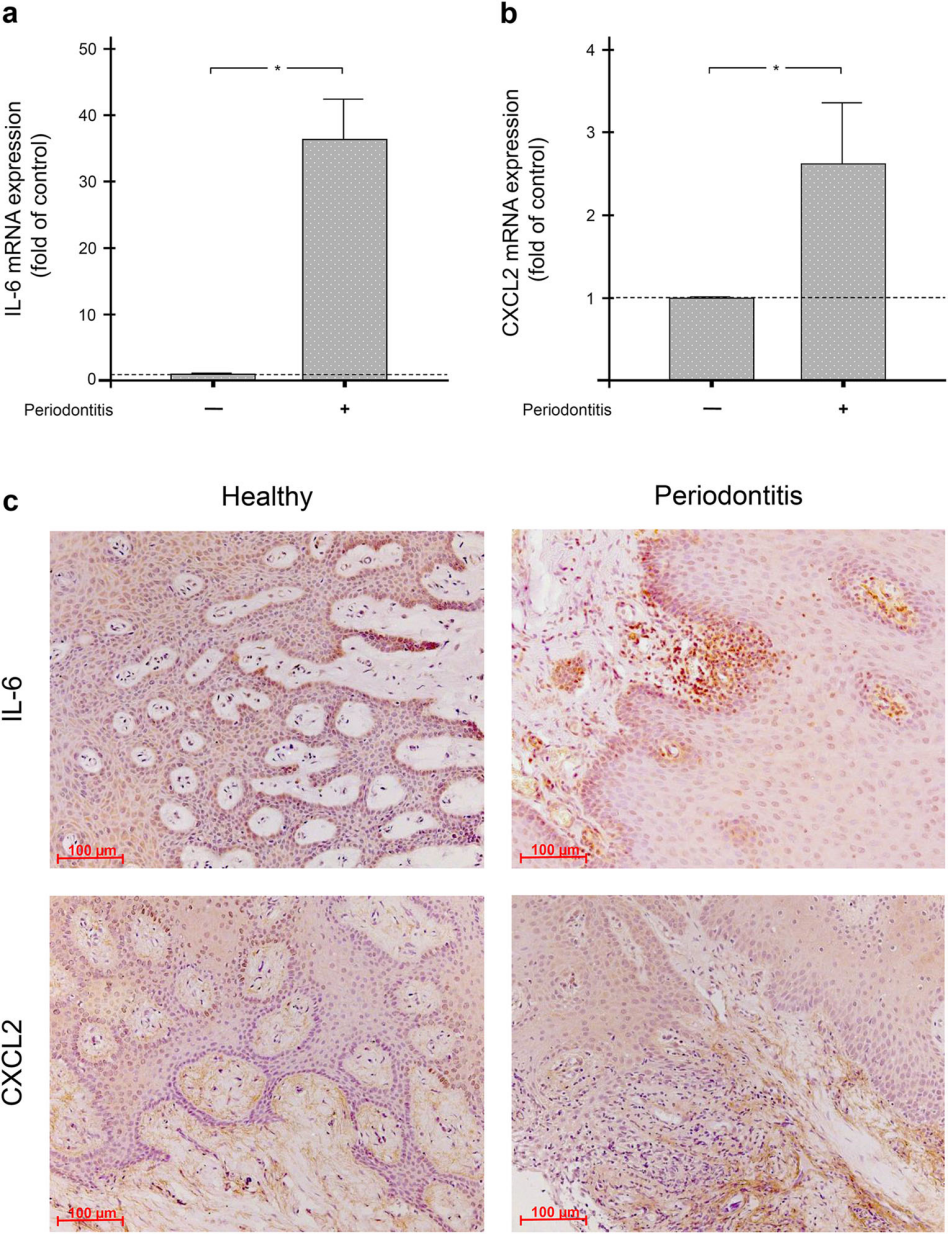
IL-6 and CXCL2 levels in human gingival biopsies. Levels of IL-6 mRNA (**a**) and CXCL2 mRNA (**b**) in gingival biopsies from periodontally healthy (n = 7) and periodontitis (n = 7) subjects, as measured by real-time PCR. *Significant (p<0.05) difference between groups. IL-6 and CXCL2 protein (**c**) in gingival biopsies from periodontally healthy and periodontitis subjects, as assessed by immunohistochemistry. Representative histological sections are shown

Since IL-6 and CXCL2 have pro-inflammatory and chemotactic activities that are increased in periodontitis, the first aim was to clarify whether IL-6 and CXCL2 are increased in gingival biopsies from periodontitis patients compared with periodontally healthy subjects. The gene expression analysis by real-time PCR revealed that the mRNA levels of IL-6 and CXCL2 were significantly (p<0.05) higher in gingival biopsies from periodontitis patients than in the gingiva of periodontally healthy individuals (Fig. 2a, b).

Whether the increased IL-6 and CXCL2 levels at the transcriptional level also exist at the protein level was then investigated by immunohistochemistry. As shown in Fig. 2c, both inflammatory mediators were detectable in the gingiva of periodontally healthy subjects. In contrast, however, immunostaining against IL-6 and CXCL2 proteins was more pronounced in the biopsies of periodontitis patients (Fig. 2c). Overall, the gene expression and immunohistochemistry analyses showed that both molecules are more abundant in gingiva during periodontal inflammation.

### IL-6 and CXCL2 levels in experimental periodontitis and/or orthodontic tooth movement in rats

**Fig. 3:**
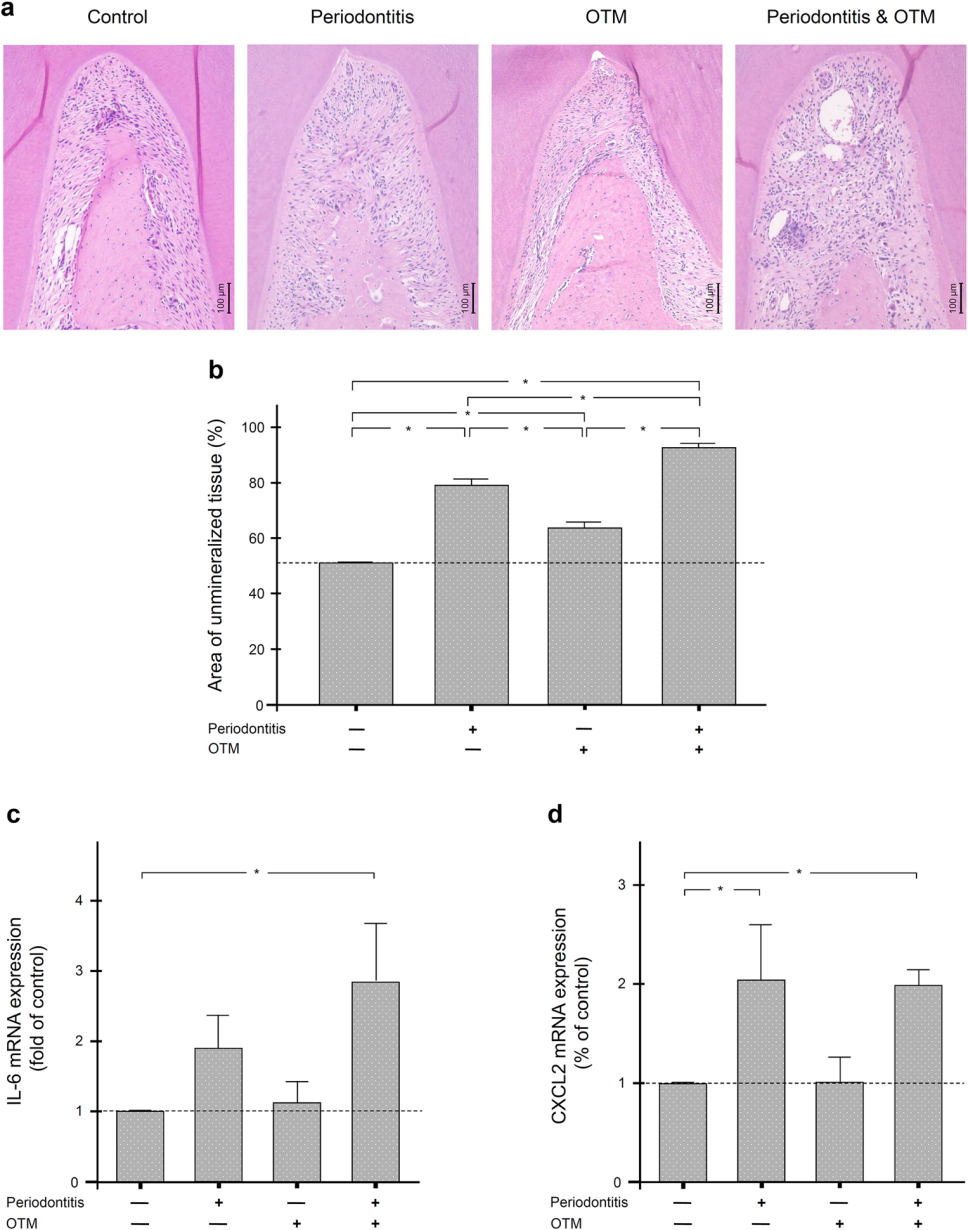
The effect of experimental periodontitis and/or orthodontic tooth movement (OTM) on alveolar bone loss as well as IL-6 and CXCL2 levels in rat gingival biopsies. Representative histological sections (**a**) and percentage area of unmineralized tissue (**b**) in the furcation region of first maxillary molars of rats in which experimental periodontitis was induced and/or OTM was performed (n = 4 rats/group). First maxillary molars from rats without periodontitis and OTM served as control. Levels of IL-6 mRNA (**c**) and CXCL2 mRNA (**d**) in gingival biopsies from first maxillary molars of rats in which experimental periodontitis was induced and/or OTM was performed (n = 4 rats/group). First maxillary molars from rats without periodontitis and OTM served as control. * Significant (p<0.05) difference between groups

To investigate the influence of orthodontic forces on increased IL-6 and CXCL2 levels in periodontitis, an animal model was used in which periodontitis was induced by ligatures and orthodontic tooth movement by a closed coil spring between the maxillary first molars and central incisors. As Fig. 3a and b reveal, the experimental periodontitis resulted in significant (p<0.05) bone loss in the furcation region of the maxillary first molars, as evidenced by an increase in unmineralized tissue in the furcation region at 8 days. Significant (p<0.05) interradicular bone loss in the furcation region was also evident when these teeth had been orthodontically moved (Fig. 3a, b). However, the most severe bone loss was observed when orthodontic tooth movement was performed in the presence of periodontitis. The differences from all other groups were significant (p<0.05) (Fig. 3a, b).

The gene expression analyses of the gingiva of rats with experimental periodontitis and/or orthodontic tooth movement also showed that periodontal inflammation was associated with an increase in IL-6 and CXCL2 levels at 8 day (Fig. 3c, d). However, this increase was only significant for CXCL2 (p<0.05). In contrast, orthodontic tooth movement alone had no significant effect on the level of either inflammatory mediator (Fig. 3c, d). The highest IL-6 gene expression level was detected when orthodontic tooth movement was performed in the presence of periodontitis (Fig. 3c). The difference from the control group was significant (p<0.05). With regard to CXCL2, orthodontic tooth movement did not lead to a further increase in CXCL2 gene expression. But here again, the difference between the combined treatment group and control was significant (p<0.05) (Fig. 3d).

### Regulation of IL-6 and CXCL2 by *F. nucleatum* and/or CTS in periodontal cells in vitro

Further experiments should then clarify whether the effects of periodontitis and/or orthodontic tooth movement on IL-6 and CXCL2 can be simulated in vitro and, if so, by what mechanism the effects of periodontitis and/or orthodontic tooth movement on these inflammatory mediators are realized.

**Fig. 4:**
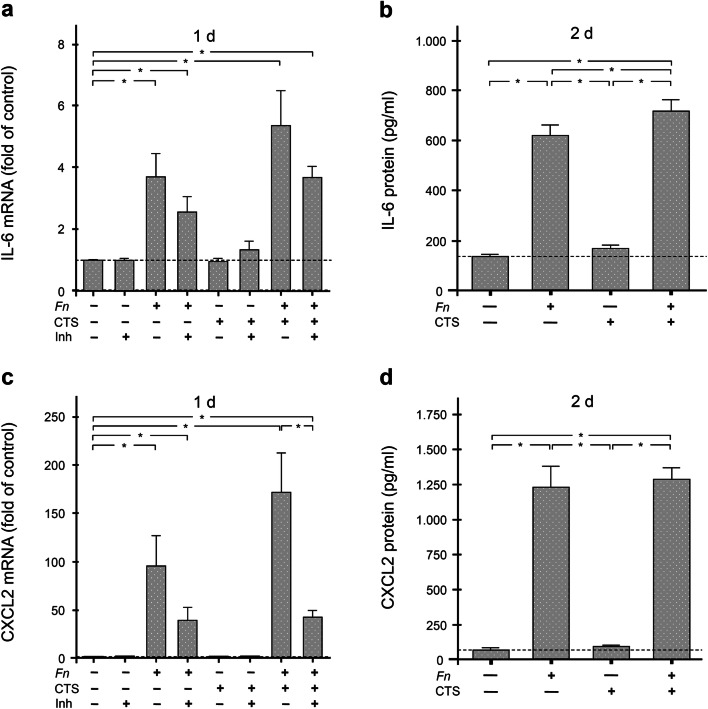
Synthesis of IL-6 and CXCL2 in response to *F. nucleatum* (*Fn*) and/or constant tensile strain (CTS) in periodontal fibroblasts. IL-6 mRNA (**a**) and protein (**b**) levels and CXLC2 mRNA (**c**) and protein (**d**) levels in cultures of periodontal fibroblasts stimulated with *F. nucleatum* (*Fn*) and/or subjected to constant tensile strain (CTS), as analyzed by real-time PCR (n = 6) and ELISA (n = 12), respectively. Untreated cells served as control. IL-6 (**a**) and CXCL2 (**c**) mRNA levels of treated and control cells, which had been pre-incubated with a specific MEK1/2 inhibitor (Inh), are also shown. * Significant (p<0.05) difference between groups

As shown in Fig. 4a, incubation of periodontal fibroblasts with *F. nucleatum* resulted in a significant (p<0.05) increase in the IL-6 gene expression, whereas application of CTS had no regulatory effect. Again, the combination of microbial and mechanical stimulation resulted in the strongest IL-6 upregulation, which was significantly different (p<0.05) from the control group but not from the *F. nucleatum* group (Fig. 4a). Preincubation of periodontal cells with the MEK1/2 inhibitor appeared to reduce the stimulatory effect of *F. nucleatum* on the IL-6 gene expressions, but this could not be statistically proven due to the high variability of cell responses. The significantly (p<0.05) stimulatory effect of *F. nucleatum* on the IL-6 gene expressions could also be confirmed at the protein level (Fig. 4b). The highest IL-6 protein level was measured when cells were exposed to both *F. nucleatum* and CTS simultaneously (Fig. 4b). The difference between the combined group and all other groups was significant (p<0.05).

Figure 4c shows that *F. nucleatum*, both alone and in combination with CTS, resulted in a significant (p<0.05) increase in the CXCL2 gene expressions. Again, CTS alone had no regulatory effect on the CXCL2 gene expression (Fig. 4c). The combination of microbial and mechanical stimulation was also able to increase the CXCL2 gene expression most strongly, with the difference from the control group again being significant (p<0.05) (Fig. 4c). However, due to the variability of cell responses, it was not possible to demonstrate a statistical difference between the *F. nucleatum* group and the combined group. Preincubation of periodontal cells with the MEK1/2 inhibitor diminished the stimulatory effect of *F. nucleatum* on the CXCL2 gene expressions, and this inhibitory effect was significant (p<0.05) in the combined group (Fig. 4c).

The significant (p<0.05) stimulatory effect of *F. nucleatum*, either alone or in combination with CTS, on CXCL2, was also confirmed at the protein level (Fig. 4d). However, there was no difference between the *F. nucleatum* and the combined group in terms of the CXCL2 protein levels (Fig. 4d).

## Discussion

In the present study, we investigated the individual and combined effects of periodontitis and orthodontic tooth movement on IL-6 and CXCL2. We were able to show that both molecules are increased in periodontal inflammation in human and rat gingival biopsies. Moreover, orthodontic tooth movement enhanced the bacteria-induced periodontal destruction and gingival IL-6 gene expression in the animal model. Furthermore, mechanical strain increased the stimulatory effect of *F. nucleatum* on IL-6 protein in vitro. Overall, our results suggest that orthodontic tooth movement can enhance bacteria-induced periodontal inflammation and destruction and that IL-6 may play a role in this process.

IL-6 is a pleiotropic member of the IL-6 family with an important role in inflammation, immune responses, osteoclastogenesis, and bone resorption as well as other processes [20, 21, 25]. Consequently, this cytokine is involved in chronic inflammatory, autoimmune and neurological diseases, cancer, and COVID-19 [20, 21, 25]. The IL-6 receptor is composed of an IL-6 binding receptor molecule (IL-6Rα) and a signal transducer, gp130. IL-6 can complex with the soluble form of IL-6Rα and thus also act on cells that only express gp130. IL-6 and sIL-6Rα contribute to the switch from acute to chronic inflammation. IL-6 triggers intracellular signaling via the Janus kinase (JAK) signal transducer and activator of transcription (STAT) and Ras Raf-mitogen-activated protein kinase (MAPK) signaling pathways [20, 21, 25]. Increased IL-6 levels can induce a further increase in IL-6 levels through positive feedback loops [20].

CXCL2 belongs to the CXC chemokine family and is synthesized by a variety of cell types, such as monocytes, macrophages, and epithelial cells in response to infection or injury [23]. CXCL2 acts as a potent chemokine for neutrophil recruitment, adhesion, and migration in inflammation. This chemokine is involved in the pathophysiology of several diseases and conditions, such as cardiovascular diseases, diabetes mellitus, and obesity [22, 23]. CXCL2 binds to its specific receptors CXCR1 and CXCR2 and triggers the p38 signaling pathway [23].

As expected, our animal model showed that periodontitis-induced bone loss can be enhanced by orthodontic tooth movement. Our findings underline that orthodontic tooth movement should only be initiated after successful periodontal therapy [26]. The present study also revealed that periodontitis is associated with increased IL-6 and CXCL2 levels in human and rat gingiva. These results are in agreement with other studies that also demonstrated increased IL-6 and CXCL2 levels in periodontitis compared to the periodontally healthy control group [27–29]. In contrast to periodontitis, orthodontic tooth movement did not increase the IL-6 and CXCL2 expressions in rat gingiva. Our results contrast with a few studies in which IL-6 and CXCL2 were increased after orthodontic tooth movement [30, 31]. The difference between our study and the other investigations could be due to the different animal models, tissue biopsies, analysis methods, and/or orthodontic tooth movement techniques. Interestingly, orthodontic tooth movement further increased the periodontitis-induced upregulation of IL-6 in our study. That orthodontic forces can further increase IL-6 under inflammatory conditions was also demonstrated by Yang et al. [32]. Whether IL-6 mediates the aggravating effect of orthodontic forces on periodontitis-induced bone loss observed in the present study should be clarified in further inhibition experiments. Interestingly, orthodontic tooth movement did not further increase the periodontitis-induced CXCL2 gene expression in rat gingiva. So far, we are not aware of any other study dedicated to the combined effect of periodontitis and orthodontic tooth movement on CXCL2.

The in vitro experiments of the present study are in close agreement with the animal experiment. The periodontal pathogenic bacterium *F. nucleatum* induced significant IL-6 and CXCL2 upregulation, whereas mechanical forces had no significant effect on the level of these two inflammatory mediators. As in animal experiments, mechanical forces were able to further increase the bacteria-induced IL-6 level. While the aggravating effect of mechanical forces on IL-6 was also observed at the protein level, such an effect was not detectable for CXCL2 protein. Preincubation of the cells with a MEK1/2 inhibitor led to a decrease in *F. nucleatum*–induced IL-6 and CXCL2 gene expressions, suggesting that *F. nucleatum* exploits, at least partially, this signaling pathway for its stimulatory effects on these inflammatory mediators. Which other intracellular signal transduction pathways *F. nucleatum* triggers in this context has to be revealed by further studies.

In our previous studies, we detected increased levels of numerous other chemokines in human and rat gingiva from sites with periodontal inflammation compared to periodontally healthy sites [18, 19]. Anti- and pro-apoptotic molecules were also elevated at sites of periodontal inflammation [17]. The animal and in vitro experiments further showed that mechanical or orthodontic forces affect the bacteria-induced levels of such molecules very differently. For example, mechanical forces inhibited the stimulatory effect of *F. nucleatum* on CXCL1, CCL2, CCL5, SOD2, and BIRC3, whereas the *F. nucleatum*–induced upregulation of CXCL5, CXCL8, and CXCL10 was enhanced [17–19]. Overall, these previous studies demonstrated that mechanical/orthodontic forces can influence the host immunoinflammatory response to periodontal bacteria. Our previous studies are thus in agreement with the present study, which clearly shows that the pro-inflammatory cytokine IL-6 is increased in the presence of periodontal pathogens and that orthodontic or mechanical forces lead to changes in the bacteria-induced IL-6 expression and synthesis. In the present study, CXCL2 was also increased in human and rat gingival biopsies of periodontitis. However, orthodontic forces had no regulatory effect on the bacterial-induced CXCL2 upregulation in rat gingiva. In vitro, mechanical forces also did not significantly alter the CXCL2 protein levels. Future studies should clarify whether other strain parameters (e.g., magnitude, duration) lead to similar or different results.

In the present study, an animal model was used because it is usually not possible for ethical reasons to systematically investigate the influence of orthodontic therapy on periodontitis in humans. Our animal model clearly showed that the ligatures on the maxillary first molars led to periodontal bone loss in the area of the furcations in these teeth. Bony remodeling processes could also be demonstrated with orthodontic tooth movement. However, as is known, these are reversible [4]. Our hypothesis that orthodontic tooth movement increases bacteria-induced periodontal destruction was confirmed. The teeth that had periodontal disease and were also moved orthodontically at the same time had the greatest bone loss, measured as a percentage of unmineralized tissue.

The number of animals included in the present study was rather small. A larger number of animals might have resulted in more significant differences. On the other hand, even with the number of animals chosen in the present study, it could be shown that the response pattern to a bacterial stimulus, alone or in combination with biomechanical forces, is very similar to the findings from the human gingival biopsies and the in vitro experiments, respectively.

To decipher the mechanism by which the effects of periodontitis and/or orthodontic tooth movement on IL-6 and CXCL2 are realized, cells were pre-incubated with a MEK1/2 inhibitor. The experiments showed that this inhibitor could reduce the *F. nucleatum*–induced upregulation of these two inflammatory mediators. However, the inhibitory effect was only statistically significant for CXCL2 in the combined group. Overall, the inhibitor experiments suggest that MEK1/2 may play an important role in bacteria-induced periodontal inflammation. Which other intracellular signaling pathways are involved in the interactions of microbial and mechanical stressors must be revealed by future studies.

In our in vitro experiments, as in some of our previous studies, periodontal fibroblasts were used [17–19, 33]. However, our immunohistochemical analysis of human and rat gingival biopsies showed that IL-6 and CXCL2 were also present in gingival epithelium. Further studies should therefore also investigate the interactions between microbial and mechanical stimuli in gingival epithelial cells and fibroblasts, dental cementoblasts, and alveolar bone cells, as well as cocultures of these cell types with immunoinflammatory cells.

In the present study, periodontal fibroblasts were incubated with *F. nucleatum*. This so-called bridging bacterium plays an important etiopathogenetic role in both gingivitis and periodontitis [12, 13]. It serves as a bridge bacterium between early and late colonizers in biofilm formation, invades periodontal cells, and supports other periodontal microorganisms to invade host cells [12, 14–16]. *F. nucleatum* was used as lysate, as in many of our previous in vitro studies [17–19]. This lysate contained lipopolysaccharides. Other virulence factors of *F. nucleatum* were certainly also present in the lysate and thus responsible for the observed stimulatory effects on IL-6 and CXCL2. Periodontitis is a multi-etiological and polymicrobial disease [34, 35]. A limitation of our study is that only the effect of *F. nucleatum* on periodontal cells was investigated in vitro. Although *F. nucleatum* is an important bacterium in the etiopathogenesis of periodontitis, *Porphyromonas gingivalis* plays a key role in the development and progression of this disease [34, 35]. Future studies should therefore also investigate the interactions of *P. gingivalis* and other periodontal pathogenic bacteria and their virulence factors, e.g., lipopolysaccharides, with biomechanical forces on IL-6 and CXCL2 in periodontal cells. Moreover, such interactions between periodontal pathogenic bacteria and mechanical forces on periodontal cells could also be investigated for other pro-inflammatory cytokines as well as anti-inflammatory mediators.

## Conclusions

Our study suggests that orthodontic tooth movement may enhance bacterially induced periodontal inflammation and destruction and that IL-6 may play a significant part in these pathogenetic processes. Our data emphasize that orthodontic tooth movement should only be performed after successful treatment of periodontitis. Furthermore, in case of periodontitis relapse, orthodontic therapy should be suspended until the periodontal inflammation has been successfully treated and thus the periodontal disease is controlled again.
